# Study on the Polymorphic Loci of Explosive Strength-Related Genes in Elite Wrestlers

**DOI:** 10.3390/genes15081068

**Published:** 2024-08-13

**Authors:** Shuo Qi, Jinglun Yu, Fanbo Meng, Zhen Wei, Zhiqiang Liang

**Affiliations:** 1 School of Sport and Health, Shandong Sport University, Jinan 250102, China; 18369668091@163.com; 2 School of Sport and Health Science, Xi’an Physical Education University, Xi’an 710068, China; lun199517@126.com; 3 School of Sports Media and Information Technology, Shandong Sport University, Jinan 250102, China; 4 The Second Clinical Medical School, Xuzhou Medical University, Xuzhou 221004, China; 5 Faculty of Sports Science, Ningbo University, Ningbo 315211, China

**Keywords:** wrestling, explosive power, single-nucleotide polymorphism, gene selection

## Abstract

This investigation aimed to explore the relationship between Chinese elite wrestlers and the polymorphic loci of explosive strength genes, and to further explore the feasibility of its application to athlete selection. The snapshot technique was used to resolve the polymorphic loci of explosive power genes in the wrestler group (59 elite wrestlers) and the control group (180 ordinary college students), and to analyze the genotype frequencies and allele frequencies of each group. A chi-square test was performed on the genotype and allele distribution data of each group to analyze the loci of explosive power genes that were associated with elite wrestlers. The loci that had an association with elite wrestlers were combined with the genotyping data, and the dominance ratios of the genotypes were calculated using the chi-square test to determine the dominant genotypes associated with elite wrestlers. The *VDR* gene rs2228570 locus exhibited statistically significant differences in genotype and allele distributions between elite wrestlers and the general population (*p* < 0.01). At the rs2228570 locus of the *VDR* gene, the difference between the CC genotype and other genotypes was statistically significant (*p* < 0.05). The rs2228570 locus of the *VDR* gene was identified as the locus associated with Chinese elite wrestlers. The polymorphism of the *VDR* gene can be used as a biomarker for Chinese wrestlers, and the CC genotype can be used as a molecular marker for the selection of Chinese elite athletes in this sport. However, expanding the sample size of elite athletes is necessary to further validate the scientific validity and feasibility of these findings.

## 1. Introduction

Wrestling is a direct-body-contact sport that constantly requires overcoming immediate changes in resistance, coupled with the high-intensity strain of confrontation sports [1]. In wrestling matches, victories and defeats are determined either by a fall (i.e., both of an opponent’s shoulders are pinned to the mat) or by accumulating points based on a wrestler’s scoring system [1]. Wrestling matches place high demands on the body’s aerobic and anaerobic energy systems [2]. The anaerobic system provides short, fast and explosive bursts of maximum strength and power, while the aerobic system helps maintain endurance throughout the match and during recovery [3,4,5]. The percentage of the body’s energy system supply in wrestling is estimated to be 60% anaerobic and 40% aerobic [1]. Anaerobic capacity is an important measure of athletic performance in elite wrestlers. Wrestling matches often include several explosive offensive and defensive moves that place high demand on the body’s anaerobic capacity [6,7,8]. Therefore, the anaerobic system is essential for wrestlers to demonstrate their technique through fast movements. Research has shown that the general physiological characteristics of elite wrestlers are higher explosive power, muscular strength, muscular endurance, aerobic capacity, flexibility, and a fat-free, moderately shaped body structure [3,8]. In other words, elite wrestlers have strong explosive power [9].

Human athletic performance is widely recognized as an individual trait dependent on the interaction between genes and environment [10]. Athletic success is influenced by a variety of genetic factors, including transcriptomic, biochemical, histological, anthropometric, physiological and psychological traits, as well as general health [11,12,13,14,15,16]. On average, 66% of the variance in athlete status can be explained by genetic factors [17]. The remaining variation is attributed to environmental factors such as deliberate practice, nutrition, performance enhancers, place of birth, availability of medical and social support, and even luck [18,19]. Exercise phenotypes, such as explosive power, endurance, muscle fiber type and proportion, and flexibility, are highly influenced by genetic factors [10,20].

Sports performance results from the combined effect of genetic factors and external environmental influences, with the impact of individual genes on sports performance currently being a major area of research worldwide [21,22,23]. In recent years, with the rapid development of gene detection technology and molecular biology, these technologies have gradually appeared to intersect, as molecular biology technology can be used to select elite athletes from a genetic perspective [24,25,26]. At present, it is not clear through which mechanisms and pathways sports genes affect the body’s athletic performance. Single-nucleotide polymorphisms (SNPs) represent only the first step in understanding the molecular basis of sport. The next decade will be a period of sports genomics with the application of new Deoxyribose Nucleic Acid (DNA) technologies for whole-genome sequencing and epigenomic, transcriptomic, and proteomic analysis [27]. Many researchers in the study of sports genes have found that such genes can determine athletic ability, affecting sports skills, and sports gene markers are one of the indicators of athlete selection that occupies the same important position as morphological indexes, physiological and biochemical indexes, indicators of special qualities, psychological indexes, and so on, and in the process of selecting athletes, the combination of genetic markers with the above corresponding indexes can increase the athlete’s success rate [24,25]. The success rate of athletes can be improved by combining the above indicators with genetic markers in the initial stages [24]. Studies have shown that genetic selection of children and adolescents during the initiation period can increase the success rate of current athletes by more than 30% [27]. Therefore, this study provides molecular markers for athlete selection by analyzing the polymorphisms of explosive strength-related loci in elite athletes.

## 2. Materials and Methods

### 2.1. Selection of Explosive Force Loci

The loci related to explosive force were identified as candidate gene loci by searching keywords such as wrestling, gene, and explosive force in PubMed, Web of Science, Sport Discus, Medline, etc. Numerous studies have explored the relationship between genetic polymorphisms and explosive power in elite athletes [28,29], including elite track and field athletes (e.g., sprinters, long jumpers, high jumpers, and throwers) and elite weightlifters [28,30,31,32,33,34]. The selection of loci for explosive strength in this study was based on the following criteria: the selected traits are associated with explosive strength, such as sprinting, long jump, weightlifting, high jump, etc., and are positively correlated with the results of studies in the literature.

(1) A literature search (1997–2024) revealed at least 128 genetic markers associated with elite athlete status, including 83 genetic markers associated with endurance and 45 genetic markers associated with explosive power; notably, among the 45 genetic markers for explosive power, the *ACE* gene rs4646994, *ACTN3* gene rs1815739, and *PPARα* gene rs4253778 were shown to be significantly associated with the status of good athletes in three or more studies [27,28,35]. Therefore, this study can include the above three loci as candidate gene loci.
(2) It has been shown that the *ACTN3* gene rs1815739, *ADRB3* gene rs4994, *CNTFR* gene rs3808871, and *VDR* gene rs7975232 loci were suggested to be correlated with the presence of explosive power in elite athletes in the study of explosive power-related gene polymorphisms and their prediction models [36,37,38]. Therefore, the *ADRB3* gene rs4994, *CNTFR* gene rs3808871, and *VDR* gene rs7975232 loci were included as a candidate loci in this study.
(3) Multiple loci are often selected for the study of the *CNTFR* gene and *VDR* gene and the results are inconsistent. To ensure the accuracy of the experimental results, the *CNTFR* gene rs41274853 and the *VDR* gene rs2228570 were included as candidate loci in this study [39,40,41,42,43,44].

In summary, the candidate loci in this study are *ACTN3* gene rs1815739, *ADRB3* gene rs4994, *CNTFR* gene rs3808871 and rs41274853, *PPARα* gene rs4253778, *VDR* gene rs7975232 and rs2228570, and *ACE* gene rs1799752.

### 2.2. Subjects and Groups

The experimental subjects in this study were divided into two groups: the wrestler group and the control group. The wrestler group represented elite wrestlers, and the control group represented healthy general college students. The wrestler group comprised elite wrestlers selected from national teams, including both international- and national-level athletes, of Han Chinese ethnicity, while the control group was randomly recruited from generally healthy college students who had no experience in professional team sports training and were of Han Chinese ethnicity.

The total number of elite wrestlers was 59, with a female-to-male ratio of 1.36:1. Therefore, when making the selection of the number of people in the general group, the male-to-female ratio of the control group should also be controlled to keep it relatively the same as that in the elite group. Relevant information points out that results that show an association in one population are difficult to replicate in another population, and the key lies in the selection of subjects; in studies of experimental and control groups, the race and gender of the two groups must be matched [27]. The ratio of men to women in the elite wrestler group in this study was kept relatively consistent with that in the control group. The sample information is shown in Table 1.

### 2.3. Moral and Ethical Review

The experimental protocol for this study was reviewed by the Expert Committee on Ethical Review, Ethical Approval No. 2019020H. Subjects were sampled using non-invasive saliva collection, and the informed consent form was read and filled out in person by the subjects before sampling.

### 2.4. Collection of DNA Samples

(1) The participants read and signed the informed consent form before sampling.
(2) Before sampling, participants rinsed their mouths with water. To avoid contamination of the samples, they did not consume any drinks or snacks after rinsing.
(3) Thirty minutes after rinsing, the testers took an oral swab for sampling and rubbed it against the inner walls of the mouth, both left and right, 10 times. After sampling, the swab was stored at 4 degrees Celsius.

### 2.5. Genotyping

The snapshot technique was used for SNP typing of the extracted DNA samples. The extension reaction system included sequencing enzymes, four kinds of fluorescent labeling of ddNTP, different lengths of extension primers next to the 5′-end of the polymorphic site, and the template of PCR products of SNP sites.

**Figure 1:**
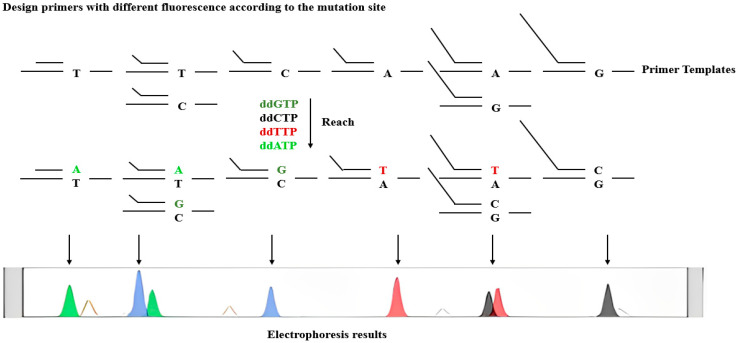
Principle of SNP experiment.

During the reaction, the primer was extended by one base and then terminated. The sequencing machine detected this, and based on the movement of the peaks, the corresponding SNP site of the extension product was determined. The color of the peaks indicated the type of incorporated base, thus determining the genotype of the sample (Figure 1).

### 2.6. PCR Primer Design and Synthesis

Primer5.0 software was used to design primers based on the gene sequences published on the NCBI website (Table 2).

### 2.7. Amplification Reaction, Extension Reaction and Purification

Refer to the Supplementary Materials for amplification reactions, extension reactions and purification steps.

### 2.8. Genotyping Results

**Figure 2:**
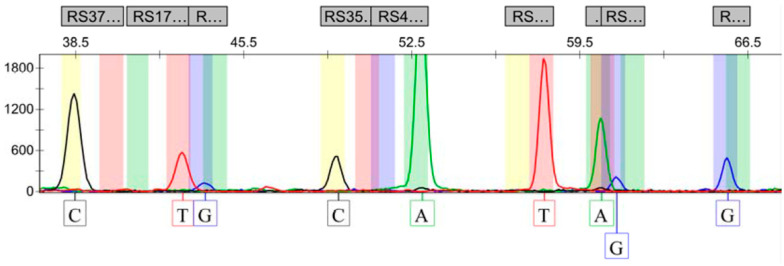
SNP typing results of random samples.

As shown in Figure 2, the presence of a single longer peak at the position corresponding to each locus indicated that the genotype of the locus was pure. In contrast, the presence of two consecutive shorter peaks indicated that the locus was heterozygous. The genotypes indicated by the random samples shown in Figure 2 were CC, TG, CC, AA, TT, GA, and GG.

### 2.9. Technology Roadmap

**Figure 3:**
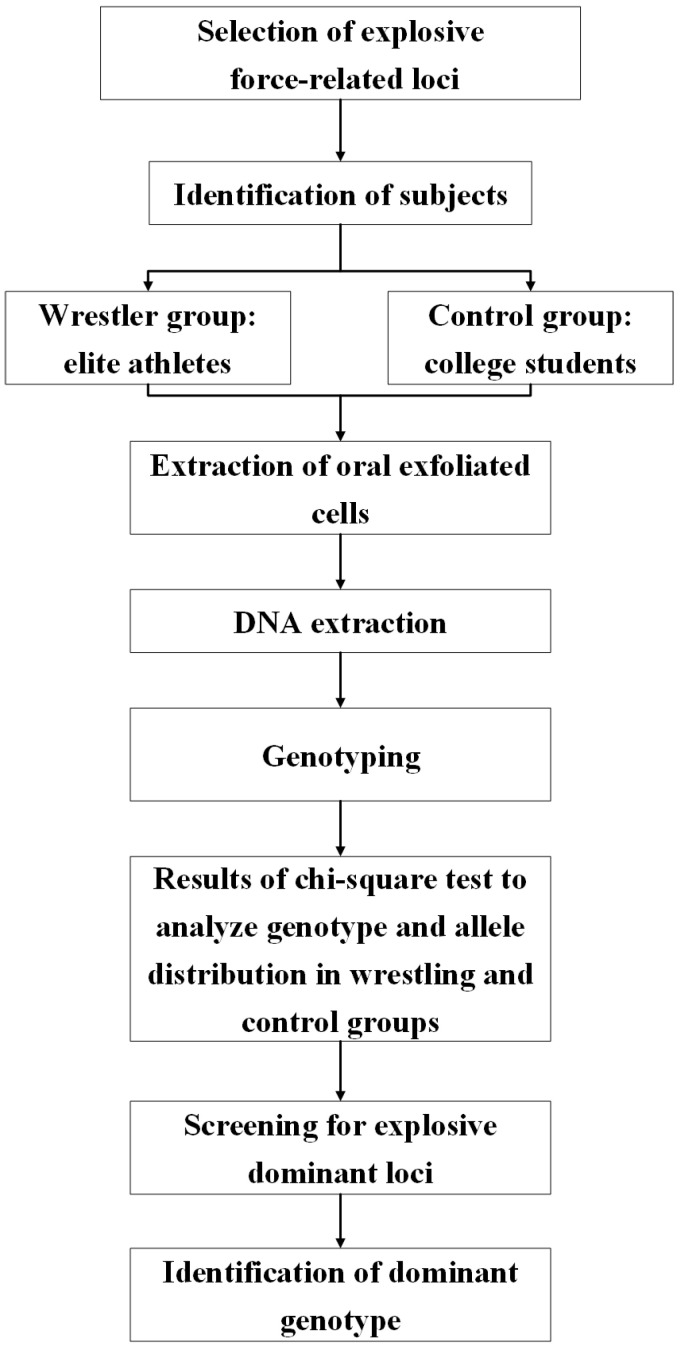
Research technology roadmap. Please refer to the Supplementary Materials for specific steps in SNP typing.

The technology roadmap for this study is shown in Figure 3.

### 2.10. Mathematical and Statistical Methods

(1) The genotyping results of each group were subjected to the Hardy–Weinberg equilibrium test using SPSS 25.0 software, and when *p* > 0.05, it means that the genotype distribution conforms to the Hardy–Weinberg equilibrium law.
(2) The genotype distribution and allele distribution data of each group were subjected to the chi-square test, and *p* < 0.05 meant that the genotype and allele distributions of the two groups were statistically significant.
(3) The genotype of each positive gene locus was determined by combining the screened positive gene loci with the genotyping data of the elite athlete group and calculating the dominance ratio of each genotype using the chi-square test.

## 3. Results

### 3.1. Results of Hardy–Weinberg Equilibrium Test Distribution for Each Site

The distribution results of the Hardy–Weinberg equilibrium test at each locus are shown in Table 3. It can be seen that the 59 elite wrestlers and 180 ordinary college students at each genetic locus conformed to the Hardy–Weinberg equilibrium test by the chi-square test, which indicated that the research subjects selected for this study were representative of the group (*p* > 0.05).

The results of the genotype distribution of candidate genes in the wrestler group and control group are shown in Table 4. The distribution of the rs1815739 locus of the *ACTN3* gene is an example. In the wrestler group, the frequency of the CC genotype was 25%, the frequency of the CT genotype was 63%, and the frequency of the TT genotype was 12%. In the control group, the CC genotype frequency was 28%, the CT genotype frequency was 52%, and the TT genotype frequency was 20%.

The results of the allele distribution of candidate loci in the wrestler group and control group are shown in Table 5. The distribution of the rs1815739 locus of the *ACTN3* gene is an example. In the wrestler group, the C allele frequency was 57%, and the T allele frequency was 43%. In the control group, the C allele frequency was 54%, and the T allele frequency was 46%.

### 3.2. Intergroup Comparison of SNP Genotype Distribution and Allele Distribution at Candidate Loci

**Figure 4:**
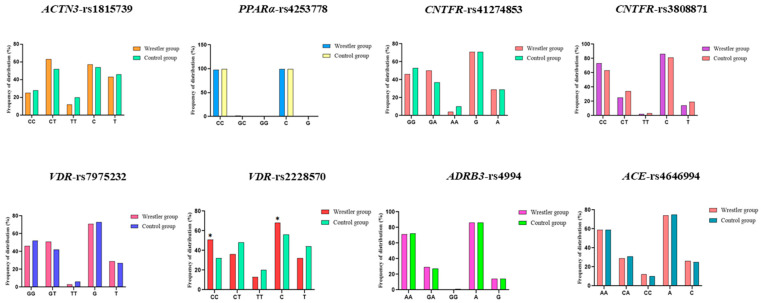
Results of genotype distribution and allele distribution of the two groups. Note: * indicates *p* < 0.05 for wrestler group vs. control group.

From the results shown in Table 6, the genotype distribution and allele distribution of the *VDR* gene rs2228570 locus differed statistically between the wrestler group and control group. As illustrated in Figure 4, the genotype frequency of CC in the wrestler group and the frequency of the C allele in the *VDR* gene rs2228570 locus were significantly higher than that of the control group. The genotype distribution and allele differences at the remaining loci were not statistically significant.

### 3.3. Calculation of the Gene Dominance Ratio at the rs2228570 Locus of the VDR Gene

A comparison of genotypic dominance ratios between groups at the rs2228570 locus of the *VDR* gene is shown in Table 7. When comparing the wrestler group with the control group, the OR of the CC genotype versus the TT genotype = 4.037 (1.007, 5.885), *p* < 0.05, and that of the CC genotype versus the (CT + TT) genotype = 2.232 (1.226, 4.065), *p* < 0.01; the OR of the CC genotype to the CT genotype = 2.155 (1.125, 4.130), *p* < 0.05. These results indicated that the CC genotype of the *VDR* gene rs2228570 locus was an advantageous genotype for explosive power compared with other genotypes, based on determining that the *VDR* gene rs2228570 locus was associated with the explosive power of elite wrestlers. It was concluded that the CC genotype of the *VDR* gene rs2228570 locus had a significant association with the explosive power of elite wrestlers. Based on determining the association between the *VDR* gene rs2228570 locus and the explosive power of elite wrestlers, it was concluded that the CC genotype was more prominent in elite wrestlers, which would make the selection of auxiliary genes more targeted in the future and improve the accuracy of selection.

## 4. Discussion

In this study, the candidate gene locus analysis method was employed. Research has shown that candidate genes can be either structural or regulatory genes [45]. The determination of candidate genes is usually carried out according to the gene that directs the synthesized protein to perform the biological function corresponding to the relevant research to find the polymorphism of the candidate gene and the correlation of the relevant phenotypic indicators [46]. Once the candidate gene is identified, relevant experiments are needed to verify the relationship with the phenotypic indicators [45].

In this study, we found that the frequency of the CC genotype at the rs2228570 locus of the *VDR* gene in elite wrestlers was significantly higher than that in the control group. The distribution frequency of the CC genotype in elite athletes was higher than the frequency of CT and TT genotypes, and the frequency of the C allele was significantly higher than that in the control group. This suggests that the polymorphic locus of this gene was associated with the elite wrestlers, and none of the remaining seven loci were associated with the elite wrestlers.

Currently, there are four loci related to the association of VDR gene polymorphisms with athletic ability: *Apa I* (rs7975232), *Bsm I* (rs1544410), *Fok I* (rs2228570), and *Taq I* (rs731236) [47]. In this study, *Apa I* (rs7975232) and *Fok I* (rs2228570) were selected as candidate loci among the above four loci. In this study, the Fok I locus was found to be associated with elite wrestlers.

The results of this study are consistent with the findings of Windelinckx et al. (2007) [48]. The difference is that the results of Windelinckx demonstrated a correlation between the Fok I locus and quadriceps muscle strength in the female population, and the quadriceps muscle strength was higher in all FF purists than in F allele carriers in females, and no correlation between this locus and phenotypic indicators was observed in males [48]. To validate these findings, further investigation involving more elite female wrestlers is necessary. One of the candidate genes recognized by researchers in recent years as being associated with improved physical strength and athlete health is the *VDR* gene [49]. VDR is a member of the nuclear receptor family [49]. Vitamin D is a fat-soluble molecule belonging to the steroid hormone family [50]. Vitamin D, synthesized in the body from cholesterol, plays a role in mechanisms affecting the structure of bone (mineral density, stress fractures, etc.) and muscle (resistance and strength), and is stored in adipocytes and transferred to the circulation when needed [51]. Vitamin D plays an important role in the maintenance of mineral homeostasis and the regulation of calcium metabolism and protein synthesis in muscle cells, and it facilitates the absorption of phosphorus and calcium by the kidneys and intestines [52]. In the metabolic function of vitamin D, functional polymorphisms on the receptor are thought to affect individuals to varying degrees. Vitamin D is essential for healthy muscle and bone development and neuromuscular function [53]. It stimulates muscle cells to receive inorganic phosphate from high-energy phosphate compounds, which play a key role in the mechanism of muscle contraction, and specific VDRs located in cell membranes enable intracellular calcium distribution and regulation [48,54]. Consequently, in the *VDR* gene rs2228570, individuals with the CC genotype have better explosive power and more advantageous neuromuscular functional connectivity.

## 5. Conclusions

The rs2228570 locus of the *VDR* gene can be identified as being associated with Chinese elite wrestlers. The polymorphism of the *VDR* gene could serve as a biomarker for Chinese wrestlers, with the CC genotype used as a molecular marker for the selection of Chinese elite athletes in this sport. However, it is still necessary to expand the sample size of elite athletes to further validate the scientific basis and feasibility of these findings.

## Figures and Tables

**Table 1:** Basic information of experimental subjects.

Group	Height (cm)	Weight (kg)	Age	Years of Training	Sample Size
Wrestler group	168.7 ± 3.5	66.7 ± 5.8	23.5 ± 2.6	9.24 ± 3.1	59 (Male 25, Female 34)
Control group	166.8 ± 4.5	59.7 ± 3.4	20.4 ± 2.5	-	180 (Male 75, Female 105)

**Table 2:** Primer names and primer sequences.

Primer Name	Primer Sequence
1-RS1815739-F	GACAGCGCACGATCAGTTCA
1-RS1815739-R	CTTGGTGTTGATGTCCTGCG
2-RS4253778-F	AATCACTCCTTAAATATGGTGGAA
2-RS4253778-R	TGATTTACCTGATGACCACCTGT
3-RS41274853-F	GAGAAATCGGATGTGAGAGGC
3-RS41274853-R	AGGAGGACCTTTTGCATTCTCT
4-RS3808871-F	CATCTGGAGGTCAAGTCCGTT
4-RS3808871-R	CCGGGATTAGACTGTGGACG
5-RS7975232-F	ATCATCTTGGCATAGAGCAGG
5-RS7975232-R	GTATCACCGGTCAGCAGTCAT
6-RS2228570-F	GGCACTGACTCTGGCTCTGAC
6-RS2228570-R	TTGCAGCCTTCACAGGTCATAG
7-RS4994-F	GCTGGGGAAGTCGCTCTCAT
7-RS4994-R	GCCAGCGAAGTCACGAACAC
8-RS1799752-F	CATCCTTTCTCCCATTTCTCTAGAC
8-RS1799752-R	CTTAGCTCACCTCTGCTTGTAAGG
1-RS1815739-F-YS	CAACACTGCCCGAGGCTGAC
2- RS4253778-R-YS	ATGGGAAATGAAGCTTTTGAATC
3-RS41274853-F-YS	AGGAGGGCCAGCTTGGTGCG
4-RS3808871-F-YS	CCCCGGTGTACCGAACCTTGC
5-RS7975232-R -YS	GGTGGGATTGAGC(A/G)GTGAGG
6-RS2228570-F-YS	CTGCTTGCTGTTCTTACAGGGA
7-RS4994-F-YS	TGGTCTGGAGTCTCGGAGTCC
8-RS1799752-R-YS	GCGAAACCACATAAAAGTGACTGTAT

**Table 3:** Chi-square test testing Hardy–Weinberg equilibrium test.

Gene Name	Locus Number	Hardy–Weinberg Balance Test			
		Wrestler Group		Control Group	
		χ^2^	*p*-Value	χ^2^	*p*-Value
*ACTN3*	rs1815739	4.55	0.10	2.23	0.33
*PPARα*	rs4253778	0.01	1.00	0.01	1
*CNTFR*	rs41274853	3.38	0.18	1.42	0.49
*CNTFR*	rs3808871	0.06	0.97	1.78	0.41
*VDR*	rs7975232	3.38	0.18	0.45	0.8
*VDR*	rs2228570	1.77	0.41	0.19	0.91
*ADRB3*	rs4994	1.67	0.43	1.12	0.57
*ACE*	rs4646994	3.87	0.14	4.01	0.13

**Table 4:** Results of genotype distribution of the two groups.

Gene Name	Locus Number	Polymorphic	Wrestler Group			Control Group		
*ACTN3*	rs1815739	C/T	CC	CT	TT	CC	CT	TT
			15 (25%)	37 (63%)	7 (12%)	50 (28%)	93 (52%)	37 (20%)
*PPARα*	rs4253778	C/G	CC	GC	GG	CC	GC	GG
			58 (98%)	1 (2%)	0 (0%)	178 (99%)	2 (1%)	0 (0%)
*CNTFR*	rs41274853	G/A	GG	GA	AA	GG	GA	AA
			27 (46%)	30 (50%)	2 (4%)	95 (53%)	67 (37%)	18 (10%)
*CNTFR*	rs3808871	C/T	CC	CT	TT	CC	CT	TT
			43 (73%)	15 (25%)	1 (2%)	114 (63%)	62 (34%)	4 (3%)
*VDR*	rs7975232	G/T	GG	GT	TT	GG	GT	TT
			27 (46%)	30 (51%)	2 (3%)	93 (52%)	75 (42%)	12 (6%)
*VDR*	rs2228570	C/T	CC	CT	TT	CC	CT	TT
			30 (51%)	21 (36%)	8 (13%)	57 (32%)	86 (48%)	37 (20%)
*ADRB3*	rs4994	A/G	AA	GA	GG	AA	GA	GG
			42 (71%)	17 (29%)	0 (0%)	130 (72%)	48 (27%)	2 (1%)
*ACE*	rs4646994	A/C	AA	CA	CC	AA	CA	CC
			35 (59%)	17 (29%)	7 (12%)	107 (59%)	57 (31%)	16 (10%)

**Table 5:** Results of allele distribution of the two groups.

Gene Name	Locus Number	Polymorphic	Wrestler Group		Control Group	
*ACTN3*	rs1815739	C/T	C	T	C	T
			67 (57%)	51 (43%)	193 (54%)	167 (46%)
*PPARα*	rs4253778	C/G	C	G	C	G
			117 (99%)	1 (1%)	358 (99%)	2 (1%)
*CNTFR*	rs41274853	G/A	G	A	G	A
			84 (71%)	34 (29%)	257 (71%)	103 (29%)
*CNTFR*	rs3808871	C/T	C	T	C	T
			101 (86%)	17 (14%)	290 (81%)	70 (19%)
*VDR*	rs7975232	G/T	G	T	G	T
			84 (71%)	34 (29%)	261 (73%)	99 (27%)
*VDR*	rs2228570	C/T	C	T	C	T
			81 (68%)	37 (32%)	200 (56%)	160 (44%)
*ADRB3*	rs4994	A/G	A	G	A	G
			101 (86%)	17 (14%)	308 (86%)	52 (14%)
*ACE*	rs4646994	A/C	A	C	A	C
			87 (74%)	31 (26%)	271 (75%)	89 (25%)

**Table 6:** Results of chi-square test for genotype distribution and allele distribution between groups.

Gene Name	Locus Number	Genotype Distribution χ^2^, *p*-Value		Allele Distribution χ^2^, *p*-Value	
		Wrestler Group vs. Control Group		Wrestler Group vs. Control Group	
*ACTN3*	rs1815739	2.91	0.233	0.36	0.549
*PPARα*	rs4253778	0.122	0.727	0.121	0.728
*CNTFR*	rs41274853	4.871	0.092	0.002	0.966
*CNTFR*	rs3808871	1.798	0.407	1.515	0.218
*VDR*	rs7975232	1.976	0.372	0.076	0.782
*VDR*	rs2228570	7.120	0.028 *	6.284	0.012 *
*ADRB3*	rs4994	0.737	0.692	0	0.992
*ACE*	rs4646994	0.769	0.113	0.736	0.769

Note: * indicates *p* < 0.05 for wrestler group vs. control group.

**Table 7:** The chi-square test for genotypic dominance.

Genotype Comparison	Wrestler Group vs. Control Group	
	OR (95% Confidence Interval)	*p*-Value
CC vs. TT	4.037 (1.007, 5.885)	0.045 *
CC vs. (CT + TT)	2.232 (1.226, 4.065)	0.008 **
CC vs. CT	2.155 (1.125, 4.130)	0.019 *

Note: * indicates *p* < 0.05 and ** indicates *p* < 0.01.

## Data Availability

The original contributions presented in the study are included in the article, further inquiries can be directed to the corresponding author.

## Supplementary Materials

The following supporting information can be downloaded at: https://www.mdpi.com/article/10.3390/genes15081068/s1, Table S1: Primer names and primer sequences, Table S2: PCR reaction system, Table S3: Temperature and time of PCR amplification system, Table S4: Digestion system for amplification product, Table S5: Extension system, Figure S1: Imaging random samples after electrophoresis.
