# Supplementary material for: Study on the Polymorphic Loci of Explosive Strength-Related Genes in Elite Wrestlers
Source: Genes (Basel). 2024 Aug 13;15(8):1068. doi: 10.3390/genes15081068 (PMC11353954; doi:10.3390/genes15081068)
Supplement: Supplementary file 1 [file genes-15-01068-s001.zip › genes-3121701-supplementary.pdf]

Extraction of DNA samples

DNA samples were extracted by using an oral swab DNA extraction kit with the following steps:

(1) Processing materials: put the wiped oral swabs in 2 ml centrifuge tubes with scissors, added 500 ul Buffer ATL and 20ul Proteinase K, vortex and mix well, and heated at 56 °C water bath for 1 hour.

(2) Sample vortexing: added 500 ul Buffer AL, vortex for 15 seconds, and heat at 70 °C for 10 minutes.

(3) Added 500 ul anhydrous ethanol, vortex for 15 seconds.

(4) Loaded HI Pure DNA Mini Column l in a 2ml collection tube, transferred to the adsorption column, and centrifuged at 10,000× *g* for 1 minute.

(5) Poured off the filtrate, placed the column back into the collection tube, transferred the remaining mixture to the adsorbent column, centrifuged at 10,000× *g* for 1 minute, and discarded the remaining filtrate and collection tube.

(6) Placed the adsorbent column in a new collection tube, added 500 ul Buffer GW1 transferred to the adsorbent column, and centrifuged at 10,000× *g* for 1 minute.

(7) Poured off the filtrate, placed the adsorbent column in a new collection tube, added 650ul Buffer GW2, and transferred to the adsorbent column, centrifuged at 10,000× *g* for 1 minute.

(8) Poured off the filtrate, put the adsorbent column back into the collection tube, and centrifuged at 10,000× *g* for 3 minutes.

(9) Placed the adsorption column in a new 1.5 ml centrifuge tube, let it dry for 5 minutes, added 20-100 ul of preheated to 70 °C Buffer AE transferred to the center of the membrane of the adsorption column, let it sit for 3 minutes, and centrifuged at 10,000× *g* for 1 minute.

(10) Stored the extracted DNA product at −20 °C to prevent DNA degradation.

Primer design

Primer5.0 software was used to design primers based on the gene sequences published on the NCBI website (Table S1).

The principles of primer design are as follows:

(1) Primer length: generally 15~30 bp, commonly used was 18~27 bp, but should not be greater than 38 bp, because too long will lead to the extension temperature being greater than 74℃, and was not suitable for Taq polymerase reaction.

(2) The efficiency of the misvocalization enzyme with A as the last base was significantly higher than the other three bases, so the use of base A at the 3′ end of the primer should be avoided, in addition, primer dimer or hairpin structure may also lead to failure of the PCR reaction.

(3) The GC content of the primer sequence was generally 40~60%, too high or too low was not conducive to the occurrence of the reaction, and the GC content of the upstream and downstream primers should not differ too much.

(4) The Tm value of the template position sequence corresponding to the primer can make the best DNA replication effect when it was around 72 °C.

(5) Primers with low ΔG (absolute value ≤ 9) and high relative valued of ΔG at the 5′ end and in the middle should be applied.

(6) High energy values of primer dimers and hairpin structures (>4.5 kcal/mol) can easily lead to the production of primer dimer bands.

Table S1. Primer names and primer sequences.

Primer name	Primer Sequence
1-RS1815739-F	GACAGCGCACGATCAGTTCA
1-RS1815739-R	CTTGGTGTTGATGTCCTGCG
2-RS4253778-F	AATCACTCCTTAAATATGGTGGAA

2-RS4253778-R	TGATTTACCTGATGACCACCTGT
3-RS41274853-F	GAGAAATCGGATGTGAGAGGC
3-RS41274853-R	AGGAGGACCTTTTGCATTCTCT
4-RS3808871-F	CATCTGGAGGTCAAGTCCGTT
4-RS3808871-R	CCGGGATTAGACTGTGGACG
5-RS7975232-F	ATCATCTTGGCATAGAGCAGG
5-RS7975232-R	GTATCACCGGTCAGCAGTCAT
6-RS2228570-F	GGCACTGACTCTGGCTCTGAC
6-RS2228570-R	TTGCAGCCTTCACAGGTCATAG
7-RS4994-F	GCTGGGGAAGTCGCTCTCAT
7-RS4994-R	GCCAGCGAAGTCACGAACAC
8-RS1799752-F	CATCCTTTCTCCCATTTCTCTAGAC
8-RS1799752-R	CTTAGCTCACCTCTGCTTGTAAGG
1-RS1815739-F-YS	CAACACTGCCCGAGGCTGAC
2- RS4253778-R-YS	ATGGGAAATGAAGCTTTTGAATC
3-RS41274853-F-YS	AGGAGGGCCAGCTTGGTGCG
4-RS3808871-F-YS	CCCCGGTGTACCGAACCTTGC
5-RS7975232-R -YS	GGTGGGATTGAGC(A/G)GTGAGG
6-RS2228570-F-YS	CTGCTTGCTGTTCTTACAGGGA
7-RS4994-F-YS	TGGTCTGGAGTCTCGGAGTCC
8-RS1799752-R-YS	GCGAAACCACATAAAAGTGACTGTAT

PCR primer design and synthesis

The PCR reaction system included buffer, primer, substrate, DNA polymerase, and ddH_2O (Table S2).

Table S2. PCR reaction system.

PCR reaction components	10 μl system
2.5 × Buffer IV	4.0
Primer（5uM，F+R）	1.5
Taq（5U/μl）	0.15
DNA（10-20ng/μl）	2
ddH_2O	2.35 μl
Total	10 μl

The amplification program is shown in Table S3.

Table S3. Temperature and time of PCR amplification system.

Temperature	Time	
95 °C	5min	
95 °C	30s	
65 °C to 55 °C	30s	10Cycles
72 °C	30s	
95 °C	30s	
55 °C	30s	30 Cycles
72 °C	30s	
72 °C	10min	

Quality control of amplification products: take 2ul of sample and electrophoresis on 2% agarose gel.

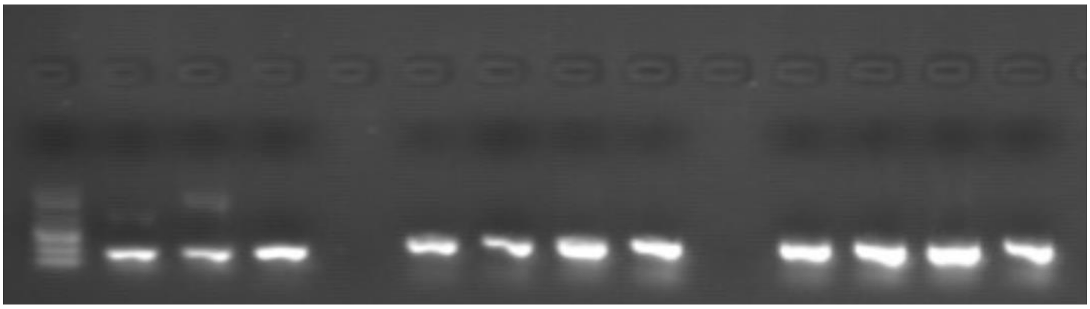

Figure S1. Imaging random samples after electrophoresis

As can be seen from Figure S1, the electrophoresis bands of the samples showed clear changes, indicating good results of the amplification products.

Amplification product digestion

For each sample, 4 ul of the amplification product was mixed in equal proportion and used for digestion. A 10-fold dilution of the ExoI enzyme resulted in the following digestion system (Table S4).

Table S4. Digestion system for amplification product.

Components	Content
PCR product	4 μl
SAP (1U/μl)	1.33 μl
ExoI (20U/μl)	(0.27μl)

Amplification product purification procedure: 37 °C for 1.0 h, 75 °C for 20 min.

Extended response

The extended reactions are shown in Table S5.

Table S5. Extension system.

Components	Content
Post-digestion amplification products	1.2 μl
PrimeRS Mix	2 ul
ABI Mix	0.5 μl
10×Buffer I	0.4 ul
H_2O	0.9 μl

Extension conditions: 96 °C for 10 s, 50 °C for 5 s, 60 °C for the 30 s, total 30 cycles.

Purification of the extension product

6 μl of the extension reaction product was added to 1 μl of CIP, 1.0 h at 37 °C and 15 min at 75 °C. The purpose of this reaction was to remove the fluorescent marker Primers Mix from the extension system.

Genotyping detection

The 3730XL sequencer was used for the detection, and the molecular weight internal standard and formamide mixture were added to each well in a 96-well plate, denatured at 95 °C, and detected on the machine.
